# The markers to delineate different phenotypes of macrophages related to metabolic disorders

**DOI:** 10.3389/fimmu.2023.1084636

**Published:** 2023-02-03

**Authors:** Quxing Wei, Yanyue Deng, Qianqian Yang, Angyu Zhan, Lexun Wang

**Affiliations:** ^1^ Guangdong Metabolic Diseases Research Center of Integrated Chinese and Western Medicine, Guangdong Pharmaceutical University, Guangzhou, China; ^2^ Key Laboratory of Glucolipid Metabolic Disorder, Ministry of Education of China, Guangzhou, China; ^3^ Guangdong Traditional Chinese Medicine Key Laboratory for Metabolic Diseases, Guangdong Pharmaceutical University, Guangzhou, China; ^4^ Institute of Chinese Medicine, Guangdong Pharmaceutical University, Guangzhou, China

**Keywords:** macrophage, molecular marker, metabolic disease, cell marker, metabolic disorders

## Abstract

Macrophages have a wide variety of roles in physiological and pathological conditions, making them promising diagnostic and therapeutic targets in diseases, especially metabolic disorders, which have attracted considerable attention in recent years. Owing to their heterogeneity and polarization, the phenotypes and functions of macrophages related to metabolic disorders are diverse and complicated. In the past three decades, the rapid progress of macrophage research has benefited from the emergence of specific molecular markers to delineate different phenotypes of macrophages and elucidate their role in metabolic disorders. In this review, we analyze the functions and applications of commonly used and novel markers of macrophages related to metabolic disorders, facilitating the better use of these macrophage markers in metabolic disorder research.

## 1 Introduction

In the last three decades, rapidly evolving technology has greatly expanded and deepened our understanding of the characteristics of macrophages. (a) Macrophages are professional phagocytes present in virtually every tissue under homeostatic physiological conditions (1). (b) Macrophages not only have important immunomodulatory functions, initiating the innate response and inflammation, but also maintain tissue homeostasis and repair (2). (c) The characteristics of macrophages are heterogeneity and plasticity, and they can be phenotypically polarized by surrounding micro-environmental stimuli (2, 3). (d) Macrophages have three distinct precursors: yolk sac (YS) macrophages, fetal liver (FL) monocytes, and bone-marrow-derived monocytes, which can be divided into two groups: tissue-resident macrophages (originating from YS and FL) and monocyte-derived macrophages (derived from bone marrow-derived monocytes) (4–6).

To study macrophage heterogeneity and plasticity, the M1/M2 dichotomy has been developed for 20 years, which offers a useful framework (7). (a) M1 macrophages play a proinflammatory role by upregulating inducible nitric oxide synthase (iNOS) to produce reactive oxygen species (ROS) and reactive nitrogen species (RNS) for activating glycolysis, fatty acid synthesis (FAS), and the pentose phosphate pathway (PPP); the M1 phenotype also suppresses the tricarboxylic acid (TCA) cycle and mitochondrial oxidative phosphorylation (OXPHOS), which promote the inflammatory response and phagocytosis. (b) M2 macrophages play an anti-inflammatory role by upregulating arginase-1 (Arg-1) to produce ornithine and urea to enhance OXPHOS, FAS, and glutamine metabolism; they also suppress PPP, thereby promoting the anti-inflammatory response and tissue repair (8, 9). However, the M1/M2 dichotomy is too simple to explain complex macrophages with various phenotypes and activation statuses in different tissues (10). Recently, cytometry and single-cell RNA sequencing have facilitated the development of macrophage marker biology. Consequently, identifying phenotypes using markers and exploring their relationship with macrophage metabolism are key points in the study of macrophages.

Metabolic diseases are noncommunicable diseases characterized by disorders of blood pressure, glucose, and lipid levels, including obesity, diabetes, hypertension, and neurodegenerative diseases (11, 12). Macrophages are important for the maintenance of homeostasis and play a profound role in the pathological state of metabolic diseases (13). (a) Obesity and diabetes: unique metabolic activation of adipose tissue macrophages (ATMs) in obesity and diabetes increases OXPHOS and glycolysis, which may be therapeutic targets to alleviate inflammation and insulin resistance (14). (b) Neurological disorders: microglia stimulated by pathological signals reprogram their metabolic pathways, such as increasing glycolysis, iron accumulation, and decreasing mitochondrial respiration, to influence neuronal functionality and survival in the brain to regulate neurological disorders (15, 16). (c) Cardiometabolic disorders: dysregulation of polarization between proinflammatory macrophages (PIMs) and anti-inflammatory macrophages (AIMs) promotes excessive inflammation and cardiac injury, resulting in cardiometabolic diseases; therefore, exploring cardiac macrophages in polarization mechanisms and their interaction with other cardiac cells is vital for future research (17).

**Figure 1:**
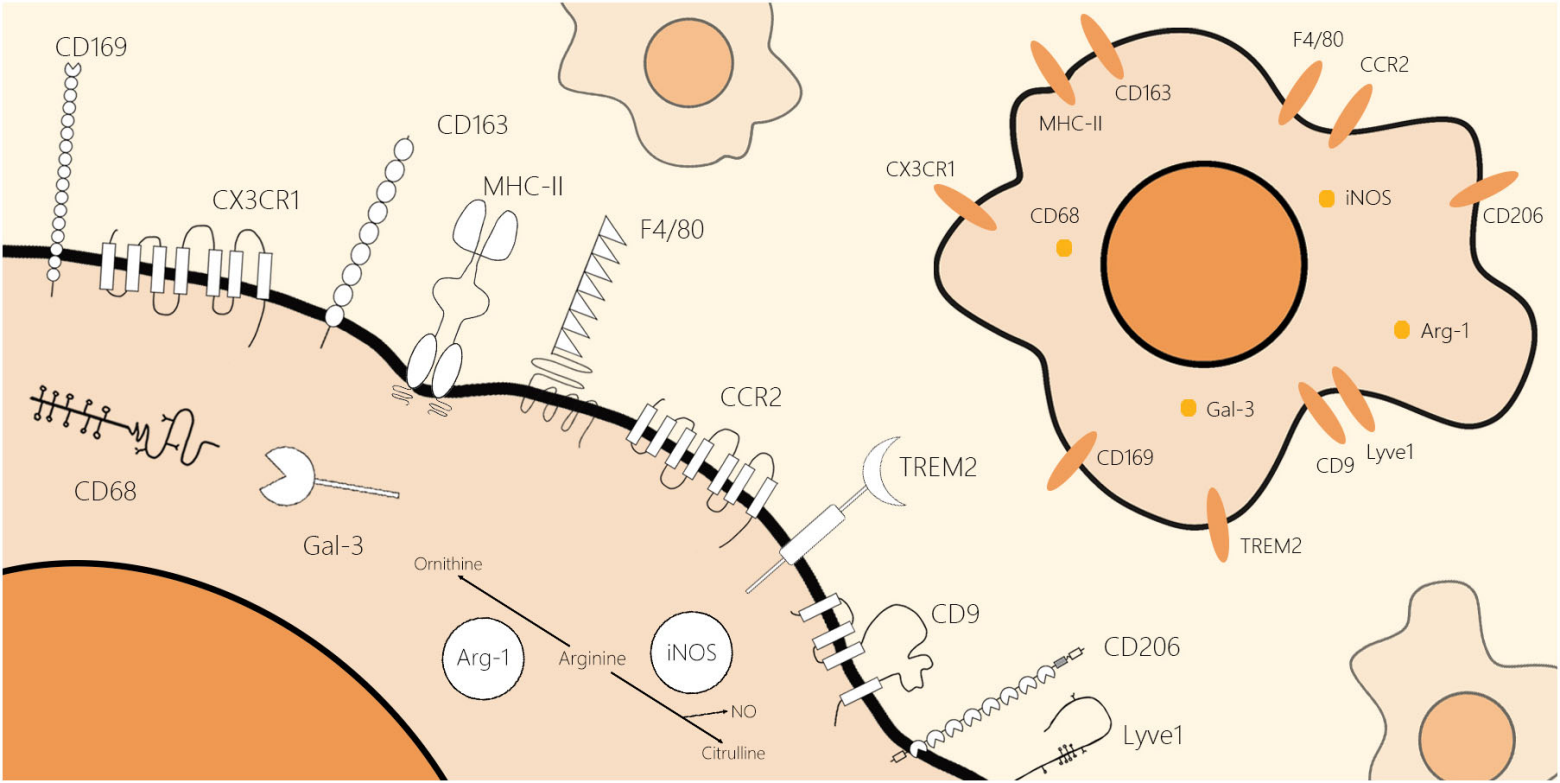
Schematic molecular structure and location of classical and novel markers of the macrophages. These markers of macrophages can be roughly divided into two categories by their location: cell surface markers and intracellular markers. F4/80, CCR2, CD169, CX3CR1, CD206, CD163, Lyve1, CD9, TREM2, and MHCII are macrophage markers located on the cell membrane. Moreover, CD68, iNOS, Arg-1, and Gal-3 are macrophage markers located inside the cell. All schematics of markers are based on their two-dimensional molecular structure.

The identification of macrophages in metabolic diseases is beneficial for the development of macrophage marker biology. In this review, we selected classical pan-markers of macrophages (F4/80 and CD68), conventional markers of macrophages in inflammatory states (iNOS and Arg-1), markers of macrophages in different tissues (C-X3-C motif chemokine receptor 1 (CX3CR1), CC chemokine receptor 2 (CCR2), lymphatic vessel hyaluronan receptor1 (Lyve1), and major histocompatibility complex class II (MHCII)), and novel markers emerging in recent years (CD9 and triggering receptor expressed on myeloid cells 2 (TREM2)), expanding on their structure and location (**Figure 1**), biological function (**Table 1**), and application in research.

**Table 1:** A list of classical and novel markers of the macrophages.

Name	Species	Functions	Expressions	Markers of macrophages	References
F4/80	Mouse	Adhesion, signaling (releasing cytokine and inducing CD8+ T cells)	Mononuclear phagocyte system	Macrophages	(18, 19)
MHCII (HLA)	Human	Antigen presentation, mediating apoptosis	Antigen-presenting cells (APCs)	Macrophages (M1, M2a, M2b)	(20, 21)
Arg-1	Human/mouse	Detoxification of ammonia in urea, wound healing, and neuroprotection	Immune cells	M2 macrophages (M2a, M2c)	(22, 23)
CCR2	Human/mouse	Mobilizing monocytes	Monocyte, macrophage	Monocyte-derived macrophage, cardiac macrophage after MI	(24, 25)
CD163	Human/mouse	Endocytosis of Hp-Hb, regulating erythropoiesis	Monocyte/macrophage lineage	Mature tissue macrophages, M2a and M2c macrophages	(26, 27)
CD169	Human/mouse	Interaction with sialoglycoconjugates, modulator of immune response	Macrophage	Macrophages in secondary lymphoid tissues	(28, 29)
CD206	Human/mouse	Binding sugar ligands, scavenging inflammatory proteins	DCs, macrophages	M2 macrophage (M2a, M2c)	(30, 31)
CD68	Human/mouse	Binding ox-LDL	Mononuclear phagocyte system	Macrophages	(32)
CD9	Human/mouse	Various physiological cellular processes	Various cells	Anti-inflammatory macrophages	(33)
CX3CR1	Human/mouse	Migration of immune cells, cytokine synthesis, cellular signaling processes, proliferation, and neuronal survival	Mononuclear phagocyte system	Tissue-resident macrophage, intestinal macrophage, microglia	(34)
Gal-3	Human/mouse	Diverse functions	Various cells	M2 macrophages	(35)
iNOS	Human/mouse	Regulating inflammation and immune response	Immune cells	M1 macrophages	(36)
Lyve1	Human/mouse	Binding and regulating hyaluronic acid, mediating leukocytes, lymphatic endothelial proliferation	Vasculature	Macrophages in vasculature	(37)
TREM2	Human/mouse	Cell activation, phagocytosis, regulation of inflammation	Myeloid lineage cells	M2 macrophages	(38)

## 2 Macrophage markers in metabolic diseases

### 2.1 Diabetes and obesity

Obesity-induced adipose tissue hypoxia promotes macrophage switching into PIMs with overexpression of iNOS (39–41). Cluster of Differentiation 9 (CD9) and TREM2 are two macrophage markers in the white adipose tissue of obese patients. Human adipocyte-related macrophages are labeled with Cluster of Differentiation 68 (CD68) and CD9 (42–44). The pan-marker CD68, proinflammatory marker iNOS, and novel markers TREM2 and CD9 are widely used markers in macrophages that are strongly associated with obesity and diabetes.

#### 2.1.1 CD68

CD68, also known as macrosialin, is a highly glycosylated transmembrane protein that is deemed a member of the scavenger receptor family because of its significant role in binding oxidized low-density lipoprotein (ox-LDL) on the macrophage cell surface (45).

CD68 is strongly expressed by the mononuclear phagocyte system, including macrophages, resulting in its use as a pan-macrophage marker (4, 46). CD68 is regarded as a marker of ATMs and has been shown to be the strongest predictor of insulin-resistant obesity (47). CD68^+^ ATMs in obese mice increased and presented a proinflammatory phenotype, which produced inflammatory cytokines, regulated glycolysis, and cleared lipids and dead adipocytes (48, 49). Similarly, CD68^+^ pancreatic islet macrophages (PLMs) are increased in diabetic patients, and their accumulation is associated with the pathogenesis of type 2 diabetes mellitus (T2DM) in humans, such as amyloid deposition (50, 51). CD68^+^ PLMs promote the compensatory proliferation of β cells and reduce glucose-stimulated insulin secretion (50, 52). In summary, CD68 is a commonly used pan-marker of tissue macrophages related to glucolipid metabolic disorder diseases, especially ATM- and PLM-mediated uptake of modified lipoproteins.

#### 2.1.2 iNOS

iNOS is widely used as a marker of PIMs because PIMs have a 30-fold higher expression of iNOS than AIMs (53). NO produced by iNOS has a significant effect on the glucolipid metabolic response. First, iNOS-derived NO in activated resident macrophages could destroy the antilipolytic effect of insulin in adipocytes, resulting in accelerated triglyceride lipolysis in adipose tissue. Second, iNOS participates in the metabolic reprogramming of immune cells to promote aerobic glycolysis in PIMs to strengthen the inflammatory response (54).

The metabolism of arginine is important in regulating macrophage polarization between pro- and anti-inflammatory subtypes because arginine is a substrate of iNOS in PIMs; in contrast, Arg-1 triggers the anti-inflammatory macrophage phenotype, competing with iNOS for arginine (55). Therefore, the future task of macrophage polarization research is to dissect the relationship between pro- and anti-inflammatory phenotypes and metabolism with respect to iNOS-derived NO.

Previously, the number of PLMs in T2DM patients with islet amyloid deposition expressing CD68 and iNOS increased, which was associated with lesion progression (56). Recent studies have shown that the downregulation of proinflammatory iNOS expression in macrophages is positively associated with improved glucose intolerance and insulin resistance after treatment (57, 58). Therefore, iNOS^+^ PIMs in obese and T2DM patients have a potent phagocytosing capacity through NO production; thus, inhibiting their formation may be a useful treatment strategy.

#### 2.1.3 CD9

CD9, also called human lymphohematopoietic progenitor cell surface antigen P24, was first identified by Kersey when combining monoclonal antibodies with acute lymphoblastic leukemia cells (59). CD9 has multiple biological functions that are involved in many vital physiological and pathological processes, such as neuroectodermal growth, myotubular formatting, and the incidence and transfer of tumors (60).

CD9 can restrict the activation of macrophages in inflammatory responses, so it is recognized as a marker for anti-inflammatory monocytes and macrophages (61, 62). However, recent studies on metabolic disorders have suggested that CD9 and TREM2 are markers of lipid-associated macrophages (LAMs) that produce proinflammatory cytokines in humans (43). In white adipose tissue, CD9^+^TREM2^+^ macrophages correlated with the severity of inflammation and influenced obesity pathology (42). In the aging brain, CD9^+^TREM2^+^ macrophages containing lipid droplets may play a pathogenic role in neurodegenerative diseases (63). Taken together, CD9 is involved in the macrophage response to lipids, and infiltrating CD9^+^ LAMs exacerbate metabolic diseases.

#### 2.1.4 TREM2

TREM2 is a lipid-sensing extracellular receptor expressed by the myeloid lineage (64, 65). TREM2 has been implicated in various biological processes, including maturation, activation, survival of cells, and regulation of inflammatory responses (65). TREM2 facilitates phagocytosis and transcription of anti-inflammatory cytokines to inhibit the production of inflammatory cytokines (66).

TREM2 was first found to be expressed on the surface of monocyte-derived dendritic cells (DCs) in humans, and it is expressed on the myeloid lineage, including macrophages (65, 67). It is expressed by a small subset of physiological tissue macrophages, such as microglia and ATMs (68). TREM2^+^ macrophages are good at lipolysis and are enriched in atherosclerotic lesions (69). TREM2 expressed by macrophages inhibits the development of metabolic disorders by facilitating cell death of prone adipocytes (43). Overall, TREM2 participates in the transmission of an inhibitory signal that reduces the inflammatory response, and TREM2^+^ macrophages are associated with lipid metabolism by suppressing lipid peroxidation and ROS to prevent systemic metabolic dysregulation.

### 2.2 Neurological disorders

Metabolic disorders trigger gut microbiota dysbiosis and low-grade systemic inflammation, leading to blood–brain barrier (BBB) dysfunction. All circulating immune cells and molecules infiltrate the brain because of increased BBB permeability, resulting in neuroinflammation and amyloid imbalance (70–72). The innate immune cells involved in this process are mainly microglia, whose classical markers are F4/80 and CX3CR1 (73–75). Recent studies have shown that M2 macrophages provide neuroprotective and regenerative effects, the targeting of which might be promising for treating chronic neurological diseases (76). In conclusion, F4/80 and CX3CR1-expressing macrophages play important regulatory roles in neuroinflammation, which polarizes to the Arg1^+^ M2 phenotype, contributing to the treatment of neurological disorders.

#### 2.2.1 F4/80

F4/80 has been extensively used in the identification and study of murine macrophages under physiological and pathological conditions since 1981, and it has greatly boosted research on macrophages (77). Extracellular epithelial growth factor (EGF) module containing mucin-like hormone receptor 1 (EMR1), the homology of F4/80 in humans, is a glycoprotein with an EGF-like domain and a seven-transmembrane motif (TM7) (78, 79). Although human EMR1 and murine F4/80 share a similar structure, EMR1 is not expressed in human macrophages but is highly expressed in eosinophils, whereas F4/80 is a well-known marker of mouse macrophages and microglia (18, 80).

F4/80 is restricted to murine macrophages in almost all tissues, including the liver, splenic red pulp, adrenal glands, and central nervous system; it may also be implicated in the generation of efferent CD8^+^ Treg cells required for inducing peripheral immune tolerance (18, 19). Nevertheless, it was later proven that apart from macrophages, F4/80 is also expressed in other myeloid cells, such as DCs (81, 82). This evidence indicates that cell specificity and limitations of F4/80 expression should be considered when labeling macrophages and microglia with F4/80.

#### 2.2.2 CX3CR1

CX3CR1 is a receptor for C-X3-C motif chemokine ligand 1 (CX3CL1) (83). CX3CL1-CX3CR1 has been shown to be a novel regulator of leukocyte transportation with adhesive and chemotactic functions; it is also a connecting bridge between neuronal cells and microglia (84). The CX3CL1-CX3CR1 axis in microglia internalizes and degrades amyloid-β deposits and Tau aggregates, which influence the development of Alzheimer’s disease (AD) (85). Hence, regulating CX3CL1 to bind microglia-expressing CX3CR1 may be a possible therapy for disease progression in the central nervous system.

CX3CR1 is widely expressed by various cells belonging to the macrophage lineage and is involved in macrophage development. For example, in the central nervous system, CX3CR1 is predominantly expressed by microglia and neurons and is engaged in activated microglia recruitment to inflammatory sites following ischemia (86). In recent years, it has served as a marker for intestinal macrophages, microglia, and patrolling monocytes (34). For instance, activated microglia were shown to have high CX3CR1 and/or MHCII expression (87). CX3CR1 can serve as a marker of tissue-resident macrophages that migrate from monocytes, especially in the brain and intestine, as well as in pathological conditions that promote inflammatory macrophages. CX3CR1^+^ microglia perform different activations to change mitochondrial dynamics and switch between OXPHOS and glycolysis, which directly affects neurological disorders.

#### 2.2.3 Arg-1

Arg-1 is a member of the ureohydrolase family of enzymes that catalyze the hydrolysis of l-arginine to urea and l-ornithine (88). The physiological functions of Arg-1 in healthy conditions include detoxification of ammonia in urea, neuroprotection, and wound healing (88, 89).

Arg-1 is a well-known marker of M2 macrophages with anti-inflammatory properties that maintain tissue homeostasis and resolve inflammation (90). A recent study has shown that promoting the polarization of microglia toward the Arg-1^+^ M2 phenotype to enhance Aβ-induced neurite atrophy and neuronal regeneration might be a therapeutic approach in the treatment of AD (91). Arg-1^+^ microglia are mainly involved in neuroprotection, and it is necessary to develop new potential drugs for neurodegenerative diseases that modulate M2 microglial polarization.

### 2.3 Cardiometabolic disorders

Metabolic disorders are major risk factors for cardiovascular disease (CVD), and metabolic syndrome increases the risk of CVD twofold (92, 93). Cardiac macrophages are identified by MHCII and CCR2, which are involved in maintaining homeostasis, immune surveillance, angiogenesis, injury repair, and assisting atrioventricular conduction (94). Recent studies have used Lyve1 and MHCII to label macrophages near blood vessels, which are involved in suppressing inflammation and fibrosis (95). Taken together, CCR2, MHCII, and Lyve1 are commonly used markers of macrophages in the cardiac system.

#### 2.3.1 CCR2

The CCR2 is a chemokine receptor, also known as the CCL2 receptor, of the monocyte chemotactic protein (MCP) family, which has a high affinity for binding (96). CCR2 engages in monocyte extravasation, adherence, and migration into inflammatory tissues, where they differentiate into macrophages (97). CCR2, antagonized by pharmacological action, can upregulate insulin sensitivity to cure obesity in mice (98). Hence, future studies on the pharmacological inhibition of CCR2 may offer guidance for therapeutic approaches in inflammatory diseases.

CCR2 has been used to label tissue macrophages originating from monocytes (99). It controls the migration of monocytes and macrophages, which play a vital role in various diseases, especially cardiometabolic disease (100). There are two distinct subsets of cardiac macrophages divided by CCR2 expression of different origins: YS-derived CCR2 macrophages and monocyte-derived CCR2^+^ macrophages (24). Over time, CCR2^−^ macrophages are progressively replaced by CCR2^+^ macrophages *via* circulation, which presents an anti-inflammatory phenotype and plays a cardioprotective role (24). Tissue-resident CCR2^+^ macrophages are vital regulators of monocyte movement, inflammation, cardiac pacemaking, and electrical propagation (101). Recent studies have expounded that the inhibition of CCR2 affects the polarization of macrophages, and the inhibition of CCL2 binding to CCR2 upregulates the expression of proinflammatory genes (102, 103). Thus, the expression and functional regulation of CCR2 in macrophages to affect inflammatory diseases need to be further investigated.

#### 2.3.2 MHCII

MHCII is a glycoprotein involved in the generation of immune responses. Its main function is to present peptide fragments from antigens to T cells to initiate an immune response (104). Moreover, MHCII also activates intracellular signaling pathways as a signaling receptor, which leads to the apoptosis of antigen-presenting cells (APCs), resulting in the termination of immune responses (105).

MHCII is expressed by innate immune cells, particularly APCs such as the monocyte-macrophage lineage (106). Macrophages expressing MHCII are activated by various inflammatory agents. IFN-γ activates macrophages to PIMs by upregulating the expression of MHCII and CD86 (20, 107). M2a macrophages induced by IL-4 are associated with increased expression of MHCII (21). IL-4 and IFN-γ both enhance MHCII expression in macrophages while also affecting macrophage polarization into different subtypes. Therefore, MHCII can be used as a pro- and anti-inflammatory marker for macrophages.

Recent studies have utilized the expression of MHCII to classify different functional macrophage subsets in metabolic diseases. In murine atherosclerosis, macrophages are subclassified into at least five subsets based on MHCII and CCR2 expression (108). In the murine heart, MHCII^hi^ macrophages are most prevalent under physiological conditions, while MHCII^low^ macrophages become the major macrophages at the early stage of myocardial infarction because MHCII expression on macrophages is transiently modulated by ischemia-reperfusion injury (109). In conclusion, macrophages with high MHCII expression play key roles in innate and adaptive immune responses and can polarize into different subtypes, thereby regulating inflammation to treat metabolic diseases by antigen presentation and inducing apoptosis.

#### 2.3.3 Lyve1

Lyve1 is a receptor of the extracellular matrix glycosaminoglycan hyaluronan located in the lymphatic endothelium (110). Lyve1 mediates the docking and transit of leukocytes, including macrophages, to influence inflammation and regulate the movement of hyaluronan, which enters peripheral lymphatics for immune activation (111–113).

Chakarov (95) demonstrated that monocyte-derived tissue macrophages could be separated into two subsets: Lyve1^low^MHCII^high^ subsets adjacent to nerve bundles and fibers and Lyve1^high^MHCII^low^ subsets near blood vessels—the latter of which played a role in inflammation influencing lung and heart fibrosis. Lim et al. (114) proved that Lyve1 expressed on perivascular macrophages interacts with hyaluronan on smooth muscle cells to protect against arterial stiffness. In conclusion, Lyve1 is mainly restricted to lymphatic endothelia and is also expressed in the liver, spleen, and lungs (111, 115, 116). Furthermore, it is expressed in a rare anti-inflammatory macrophage subset with a potent endothelial progenitor appearing in inflammatory and tumor sites (116–118). Lyve1^+^ macrophages interact with hyaluronan, influencing endothelial junctional retraction and proliferation. Future research should investigate the relationship between macrophages in the lymphatic and vascular systems and Lyve1.

## 3 Macrophages in metabolic tissues

Since these markers are expressed but not restricted to certain tissues, macrophages identified by these markers distribute in virtually all tissues related to metabolic disorders. For instance, TREM2^+^ macrophages resided in different tissues during obesity, including adipose tissue and liver, with different phenotypes, such as LAMs, non-alcoholic steatohepatitis-associated macrophages, and scar-associated macrophages (119). Identifying various macrophage phenotypes requires the combination of multiple markers, which helps to visualize macrophage heterogeneity and plasticity in affecting the metabolic microenvironment of tissues. For example, glucose uptake and utilization are upregulated in cardiac macrophages (classified by TIMD4, Lyve1, MHCII, and CCR2), and free fatty acids promote dysregulation of polarization, resulting in excessive inflammation, activation of myofibroblast, and apoptosis of cardiomyocytes during the metabolic disorders (120, 121). This evidence proves macrophages identified by various markers in metabolic tissues may be therapeutic targets to alleviate inflammation and insulin resistance.

## 4 Conclusion

Macrophages are critical immune cells located in various tissues and are polarized to various phenotypes depending on the tissue microenvironment. Macrophage polarization influences metabolism by regulating inflammation in metabolic diseases. F4/80 is a pan-marker of mouse macrophages and microglia, whereas CD68 is a pan-marker of human and mouse macrophages. iNOS has been identified as a proinflammatory marker, Arg-1 and CD9 are anti-inflammatory markers, and MHCII is a marker of both states. CD9 and TREM2 are novel markers that are associated with glucose and lipid metabolism, respectively. CX3CR1 is expressed on microglia, Lyve1 on macrophages of the vasculature, and CCR2 and MHCII on cardiac macrophages. Applying a combination of origin, recruitment dynamics, physiological and pathological functions, and marker expression to defined macrophage phenotypes may be a new approach to investigating macrophages in metabolic disorders.

## Author contributions

QW: conceptualization, writing–Original draft preparation, and visualization. LW: conceptualization, supervision, and project administration. YD: writing—original draft preparation. QY: resources. AZ: investigation. All authors contributed to the article and approved the submitted version.
